# Loss-of-function mutations in *CFAP57* cause multiple morphological abnormalities of the flagella in humans and mice

**DOI:** 10.1172/jci.insight.166869

**Published:** 2023-02-08

**Authors:** Ao Ma, Jianteng Zhou, Haider Ali, Tanveer Abbas, Imtiaz Ali, Zubair Muhammad, Sobia Dil, Jing Chen, Xiongheng Huang, Hui Ma, Daren Zhao, Beibei Zhang, Yuanwei Zhang, Wasim Shah, Basit Shah, Ghulam Murtaza, Furhan Iqbal, Muzammil Ahmad Khan, Asad Khan, Qing Li, Bo Xu, Limin Wu, Huan Zhang, Qinghua Shi

**Affiliations:** 1 Division of Reproduction and Genetics, First Affiliated Hospital of University of Science and Technology of China (USTC), Hefei National Laboratory for Physical Sciences at Microscale, School of Basic Medical Sciences, Division of Life Sciences and Medicine, Biomedical Sciences and Health Laboratory of Anhui Province, University of Science and Technology of China, Hefei, China.; 2 Institute of Pure and Applied Biology, Zoology Division, Bahauddin Zakariya University, Multan, Pakistan.; 3 Gomal Centre of Biochemistry and Biotechnology, Gomal University, Dera Ismail Khan, Pakistan.; 4 The Central Laboratory of Medical Research Center, First Affiliated Hospital of USTC, Hefei, China.

**Keywords:** Reproductive Biology, Fertility, Genetic diseases, Mouse models

## Abstract

Multiple morphological abnormalities of the sperm flagella (MMAF) are the most severe form of asthenozoospermia due to impaired axoneme structure in sperm flagella. Dynein arms are necessary components of the sperm flagellar axoneme. In this study, we recruited 3 unrelated consanguineous Pakistani families with multiple MMAF-affected individuals, who had no overt ciliary symptoms. Whole-exome sequencing and Sanger sequencing identified 2 cilia and flagella associated protein 57 (*CFAP57*) loss-of-function mutations (c.2872C>T, p. R958*; and c.2737C>T, p. R913*) recessively segregating with male infertility. A mouse model mimicking the mutation (c.2872C>T) was generated and recapitulated the typical MMAF phenotype of *CFAP57*-mutated individuals. Both *CFAP57* mutations caused loss of the long transcript-encoded CFAP57 protein in spermatozoa from MMAF-affected individuals or from the *Cfap57*-mutant mouse model while the short transcript was not affected. Subsequent examinations of the spermatozoa from *Cfap57*-mutant mice revealed that CFAP57 deficiency disrupted the inner dynein arm (IDA) assembly in sperm flagella and that single-headed IDAs were more likely to be affected. Thus, our study identified 2 pathogenic mutations in *CFAP57* in MMAF-affected individuals and reported a conserved and pivotal role for the long transcript-encoded CFAP57 in IDAs’ assembly and male fertility.

## Introduction

Infertility is a global human health concern, affecting approximately 15% of couples (1). Male factors account for approximately half of infertility cases (2). One of the major causes of male infertility is asthenozoospermia, which is defined by the World Health Organization (WHO) as a reduction in sperm motility or progressive motility (3). Multiple morphological abnormalities of the sperm flagella (MMAF) are the most severe form of asthenozoospermia, with absent, short, bent, coiled, and irregular-caliber flagella (4). The genetic causes of MMAF have been widely studied in male infertility (5). To date, whole-exome sequencing (WES) studies have identified 36 genes causing MMAF in humans, including cilia and flagella associated protein 43 (*CFAP43*), *CFAP44*, *CFAP47*, *CFAP61*, *CFAP65*, *CFAP69*, *CFAP70*, *CFAP91*, *CFAP251*, and dynein axonemal heavy chain 17 (*DNAH17*) after being mutated (6–24). However, only approximately 60% of MMAF cases can be explained by current genetic findings (12), which emphasizes the need for further research into the genetic factors involved in MMAF.

Motile cilia and flagella, both evolutionarily conserved organelles, possess a highly organized structure arranged as a 9 + 2 arrangement of 9 microtubule doublets (MTDs) surrounding a pair of singlets (the central pair of microtubules), with radial spokes extending from each of the peripheral doublets toward the central pair (25, 26). Inner dynein arms (IDAs) and outer dynein arms (ODAs) are protein complexes that attach to peripheral MTDs. They are responsible for the cilia or flagella beating through the hydrolysis of ATP (25). Disease-causing mutations have been identified in genes encoding IDA and ODA components in men with MMAF, including *DNAH1*, *DNAH2*, *DNAH6*, *DNAH8*, *DNAH10*, and *DNAH17* (4, 10, 22, 27–29). Meanwhile, mutations in these genes could also cause primary ciliary dyskinesia (PCD), which is characterized by chronic obstructive pulmonary disease, reduced fertility or asthenozoospermia, randomization of a left-to-right body axis, and other symptoms, due to defects in the conserved structure of motile cilia (30–34). Defects in the proteins responsible for the assembly of IDAs or ODAs can also lead to MMAF and PCD (12, 35). However, very few of these protein-encoding gene mutations have been identified in PCD- or MMAF-affected individuals and the corresponding animal model studies.

In this study, we recruited 3 unrelated consanguineous Pakistani families with 7 infertile men, 4 of whom were diagnosed with asthenozoospermia. WES and Sanger sequencing identified 2 homozygous *CFAP57* stop-gain mutations (c.2872C>T, p. R958* in family 1; c.2737C>T, p. R913* in family 2 and family 3) cosegregating with the MMAF phenotype in all 3 families. We generated *Cfap57*-mutant mice and found that mutant male mice were infertile due to MMAF. Both *CFAP57* mutations caused loss of the long transcript-encoded CFAP57 protein in spermatozoa from MMAF-affected individuals. Further studies showed that the defect in *Cfap57*-mutant sperm flagella was caused by abnormal assembly of IDA proteins, of which the single-headed IDA protein was the most severely affected. Collectively, these findings demonstrate that loss-of-function mutations in *CFAP57* caused MMAF and male infertility in mice and humans, providing evidence for an essential and specific role for long transcript-encoded CFAP57 in male reproduction.

## Results

### MMAF-affected individuals from 3 unrelated consanguineous Pakistani families.

**Figure 1:**
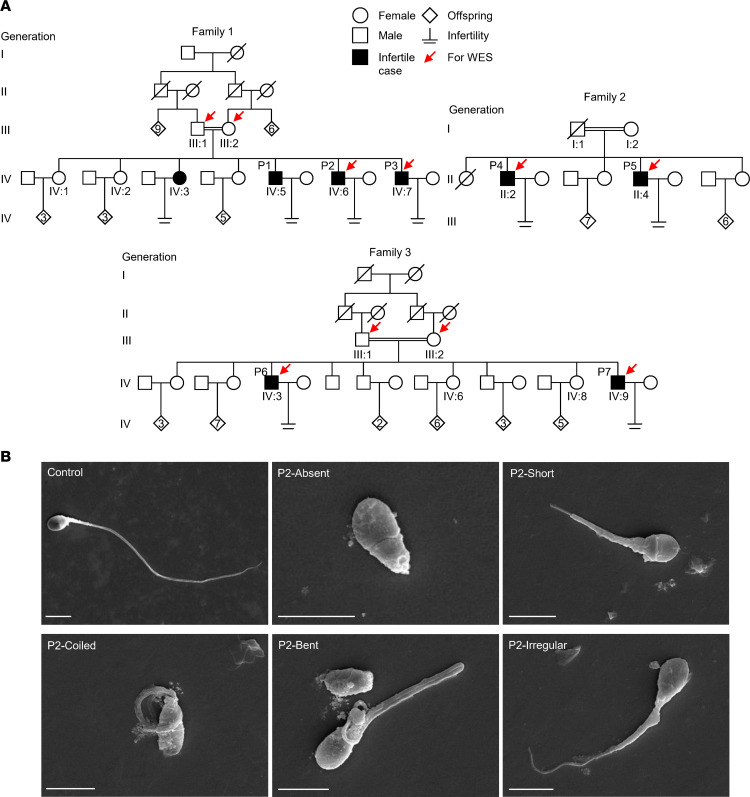
Affected individuals from 3 unrelated consanguineous Pakistani families. (**A**) Pedigrees of family 1 with 4 infertile cases, P1 (IV:5), P2 (IV:6), and P3 (IV:7); family 2 with 2 infertile cases, P4 (II:2) and P5 (II:4); and family 3 with 2 infertile cases, P6 (IV:3) and P7 (IV:9). Arrows point to the individuals for which WES was performed. Slashes denote deceased family members, and double horizontal lines represent consanguineous marriages. (**B**) Representative SEM micrographs showing sperm morphological abnormalities observed in P2, including absent, short, coiled, bent, and irregular-caliber flagella. A representative spermatozoon with normal morphology from the fertile control was shown. Scale bars represent 5 μm.

Three unrelated consanguineous Pakistani families with multiple infertility-affected individuals were recruited for this study. There were 3 infertile brothers in family 1, P1 (IV:5, 27 years old), P2 (IV:6, 36 years old), and P3 (IV:7, 42 years old); 2 infertile brothers in family 2, P4 (II:2, 59 years old) and P5 (II:4, 53 years old); and 2 infertile brothers in family 3, P6 (IV:3, 49 years old) and P7 (IV:9, 36 years old) (Figure 1A). Despite having regular unprotected sexual intercourse for at least 8 years, all of these individuals experienced infertility. Genetic factors known to cause male infertility, such as azoospermia factor microdeletions and abnormal karyotypes, have been ruled out. All available clinical characteristics are listed in Table 1. P2, P5, P6, and P7 were diagnosed with asthenozoospermia, and P3 was diagnosed with azoospermia. The morphology of spermatozoa from P2 and P7 was analyzed using H&E staining of semen smears, and the results revealed that most spermatozoa from the 2 individuals had malformed flagella, including absent, short, coiled, bent, and irregular caliber, which are typical characteristics of MMAF (Table 1 and Supplemental Figure 1; supplemental material available online with this article; https://doi.org/10.1172/jci.insight.166869DS1). The scanning electron microscopy (SEM) analysis results of spermatozoa from infertile cases further demonstrated the presence of MMAF (Figure 1B). In addition, P6 had orally told that he had typhoid, headache, and difficulty in breathing sometimes but refused to participate in any further examinations. All the other 6 affected individuals claimed they were not experiencing any other PCD-related symptoms, except infertility.

### Biallelic mutations in CFAP57 identified in the affected individuals.

**Figure 2:**
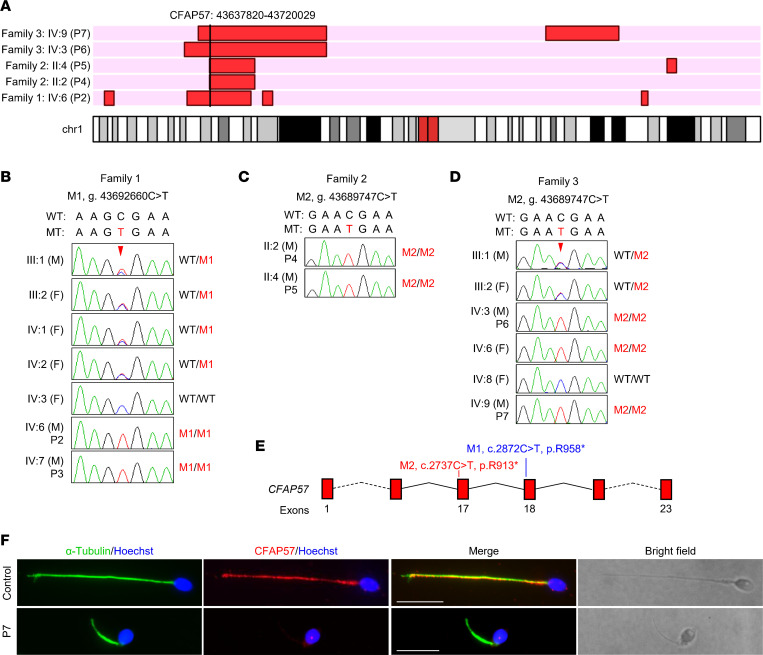
*CFAP57* loss-of-function variants identified in MMAF cases. (**A**) *CFAP57* variants are located in the regions of homozygosity (RoHs) of affected individuals. RoHs are marked in red. (**B**–**D**) Verification of the *CFAP57* variants, p.R958* (NC_000001.10:g.43692660C>T) in family 1 (**B**) and p.R913* (NC_000001.10:g.43689747C>T) in family 2 (**C**) and family 3 (**D**), by Sanger sequencing using genomic DNA from all the available family members. Arrowheads, the mutation sites; F, female; M, male; WT, wild-type allele; MT, the mutant allele; M1, c.2872C>T; M2, c.2737C>T. (**E**) The positions of the 2 variants in *CFAP57* at transcript level (NM_001195831.2) and protein level (NP_001182760.2). (**F**) Representative images of spermatozoa from a fertile control and P7 costained by anti–α-tubulin antibodies and anti-CFAP57 antibodies. Scale bars represent 10 μm.

To investigate the genetic causes of MMAF, we conducted WES analyses in all 3 families, including P2 and his parents from family 1; P4 and P5 from family 2; and P6, P7, and their parents from family 3 (Figure 1A). Through variant screening, we identified 2 homozygous stop-gain mutations in *CFAP57* (M1: NM_001195831.2, c.2872C>T, p.Arg958* in family 1, M2: c.2737C>T, p. R913* in families 2 and 3) as candidate MMAF-causing mutations. Both *CFAP57* mutations were located in the regions of homozygosity (RoHs) of affected individuals (Figure 2A and Supplemental Figure 2). Subsequent Sanger sequencing of all the available family members demonstrated that *CFAP57* mutations were recessively segregated with the MMAF phenotype in all the family members (Figure 2, B–D). Both mutations had extremely rare frequencies in large genome data sets from the 1000 Genomes Project and Genome Aggregation Database (Supplemental Figure 2 and Supplemental Table 1). Both *CFAP57* mutations were predicted to be highly deleterious by computational software, including MutationTaster (36), fathmm-MKL (37), GERP++ (38), and SiPhy (39) (Supplemental Figure 2 and Supplemental Table 1). As recorded in the NCBI Gene database, there are 2 *CFAP57* transcript isoforms in humans (NM_001195831.2 and NM_152498.3), encoding 2 CFAP57 proteins with 1,250 aa and 698 aa, respectively. Reverse transcription polymerase chain reaction (PCR) revealed that both transcripts of *CFAP57* existed in human testes and nasal epithelial cells (Supplemental Figure 3, B–D). The M1 and M2 we identified only affect the long isoform of *CFAP57* (NM_001195831.2) (Supplemental Figure 3A).

Considering that both *CFAP57* mutations were predicted to introduce a premature stop codon, which may result in mRNA degradation or truncated proteins (Figure 2E), we generated an antibody targeting the long isoform of the human CFAP57 amino acids (aa) 701 to 900 to detect whether the predicted truncated proteins were present. The specificity of this antibody was verified, as it could detect human or mouse CFAP57 protein overexpressed in HEK293T cells (Supplemental Figure 4). Afterward, we performed immunostaining with spermatozoa of P7 (harboring homozygous M2) with this antibody. The α-tubulin signals were normal compared to those of the control spermatozoa, while the CFAP57 signals were totally absent in P7 spermatozoa (Figure 2F). Thus, the *CFAP57* mutation M2 caused a total loss of CFAP57 in sperm flagella from P7.

### Cfap57-mutant mice recapitulate the MMAF phenotype of individuals with homozygous CFAP57 mutations.

**Figure 3:**
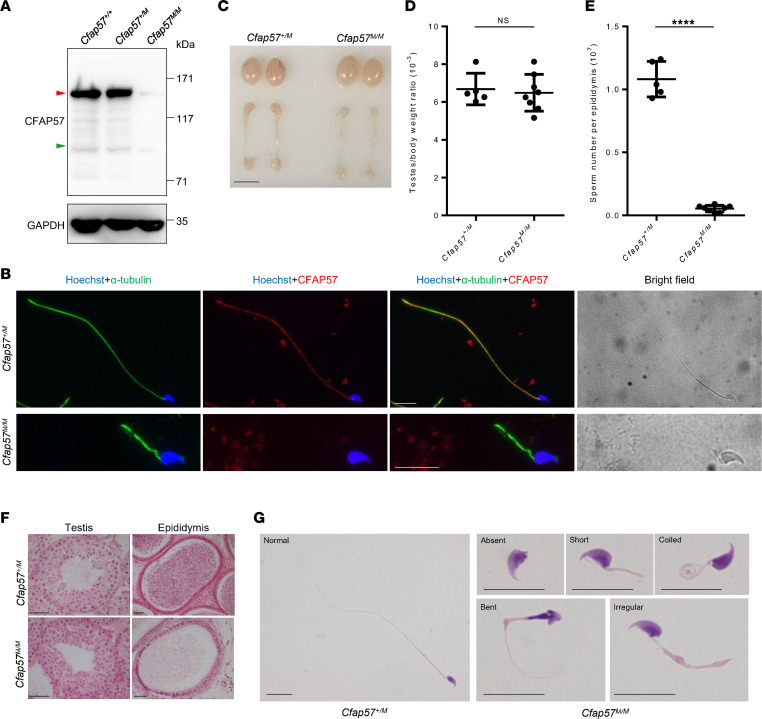
*Cfap57^M/M^* mice exhibit typical MMAF phenotype. (**A**) Immunoblotting with lysates of testes using anti-CFAP57 antibody. GAPDH was used as the loading control. The red arrow points to the full length of CFAP57, and the green arrow points to the predicted truncated protein. (**B**) Representative images of spermatozoa from *Cfap57^+/M^* and *Cfap57^M/M^* mice costained with anti–α-tubulin and anti-CFAP57 antibodies. Scale bars represent 10 μm. (**C**) Representative images of testes and epididymides from *Cfap57^+/M^* and *Cfap57^M/M^* mice. The scale bar represents 5 mm. (**D**) Testes/body weight ratio of *Cfap57^+/M^* and *Cfap57^M/M^* mice. (**E**) Spermatozoa from 1 epididymis were counted for *Cfap57^+/M^* and *Cfap57^M/M^* mice. (**F**) Representative images of testis and epididymis sections of *Cfap57^+/M^* and *Cfap57^M/M^* mice after H&E staining. Scale bars represent 50 μm. (**G**) Representative images of spermatozoa after H&E staining showing normal flagella from *Cfap57^+/M^* mice and absent, short, coiled, and bent flagella and flagella of irregular caliber from *Cfap57^M/M^* mice. At least 200 sperm were examined. Scale bars represent 10 μm. In all the above experiments, 8-week-old mice were used. ****P < 0.0001 by unpaired Student’s 2-tailed *t* test.

In order to determine the effect of the identified M1 mutation, we generated *Cfap57*-mutant (*Cfap57^M/M^*) mice (NM_026789.5, c.2865-2871del, p. Lys956Phefs*4) to imitate the M1 mutation identified in family 1 (Supplemental Figure 5A). In mice, we also detected the presence of a long isoform and a short isoform, homologous to the 2 isoforms in humans, in mouse testes and trachea (Supplemental Figure 3, E–G). The *Cfap57* mutation is predicted to produce a truncated long isoform, with the short isoform unaffected. Immunoblotting using the CFAP57 antibody that specifically recognized the protein segment encoded by the long transcript (701 to 900 aa) did not detect the WT and truncated CFAP57 proteins in *Cfap57^M/M^* mouse testes (Figure 3A). Consistently, immunofluorescence staining did not show CFAP57 signals in the sperm tail in *Cfap57^M/M^* mice (Figure 3B). These results indicate the absence of wild-type or truncated long isoforms in the mouse model, imitating the effect of the M1 mutation identified in family 1.

*Cfap57^M/M^* mice showed normal growth and development. *Cfap57^M/M^* male mice were infertile, with normal testicular size and testes/body weight ratio compared to *Cfap57^+/M^* mice (Figure 3, C and D). However, sperm number was significantly reduced in *Cfap57^M/M^* mouse epididymides, which was only approximately 5% of *Cfap57^+/M^* mice (Figure 3E). The numbers of spermatids and spermatozoa in the testes and epididymides were markedly reduced in *Cfap57^M/M^* mice (Figure 3F). *Cfap57*-mutant spermatozoa were all morphologically abnormal with a typical human MMAF-like phenotype (Figure 3G and Table 2) and were immotile according to computer-aided sperm analysis (CASA) (Table 2 and Supplemental Videos 1 and 2). Hence, *Cfap57^M/M^* mice recapitulated the MMAF phenotype found in the affected individuals carrying homozygous M1 mutations, showing that the *CFAP57* mutation is indeed pathogenic for MMAF in both infertile cases and mutant mice.

### Single-headed IDAs are affected after Cfap57 mutation.

**Figure 4:**
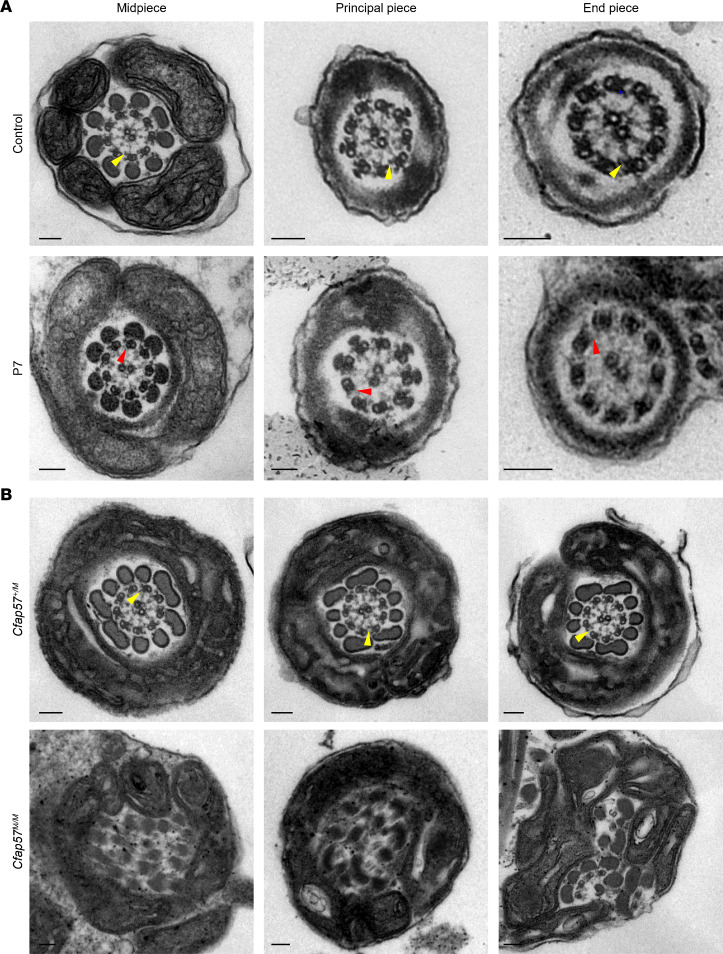
Loss of IDA in spermatozoa from the infertile case and *Cfap57^M/M^* mice. (**A**) Representative TEM micrographs showing cross sections of midpiece, principal piece, and end piece of sperm flagella from a fertile control and P7. (**B**) Representative TEM micrographs showing cross sections of sperm flagella from *Cfap57^+/M^* and *Cfap57^M/M^* mice. The yellow triangle marks the IDAs, and the red triangle marks the position where IDAs are absent. Scale bars represent 100 nm.

To explore how *CFAP57* mutations cause MMAF in humans and mice, the ultrastructure of spermatozoa from P7 and *Cfap57^M/M^* mice was investigated. The axoneme structure in P7’s spermatozoa had a similar 9 + 2 arrangement to that of a fertile control. Detailed analysis of dynein arms revealed that the IDAs were absent, while ODAs were identifiable in the patient (Figure 4A). Sperm of *Cfap57^M/M^* mice exhibited more severe abnormalities, with all the analyzed cross sections showing a disorganized axoneme structure (Figure 4B).

**Figure 5:**
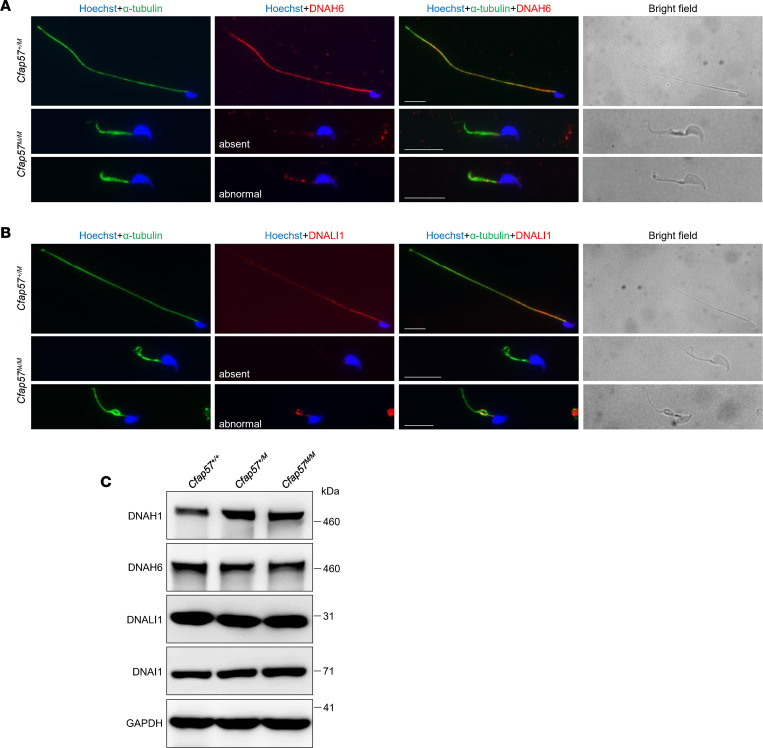
CFAP57 is required for the assembly of IDAs. (**A** and **B**) Representative images of spermatozoa from *Cfap57^+/M^* and *Cfap57^M/M^* mice costained by anti–α-tubulin and anti-DNAH6 antibodies (**A**) or anti-DNALI1 antibodies (**B**). Scale bars represent 10 μm. (**C**) Immunoblotting with lysates of testes using anti-DNAH1, anti-DNAH6, and anti-DNALI1. DNAI1 and GAPDH were used as the loading controls.

To determine the structural basis of axoneme abnormalities, immunofluorescence staining of the IDA markers (DNAH10, DNAH6, and DNALI1) and ODA markers (DNAI1, DNAI2, DNAH8, and DNAH17) along with a microtubule marker (α-tubulin) in sperm from *Cfap57^M/M^* mice was performed. In control mice, the signals of α-tubulin and IDA and ODA proteins were detected along the entire flagella of spermatozoa. In *Cfap57^M/M^* mice, the α-tubulin signals could be observed along the flagella of sperm. ODA proteins were present on the mutant sperm flagella, though showing a discretely distributed pattern (Supplemental Figure 6, A–D), while signals of DNAH6 and DNALI1 (single-headed IDAs) were either absent (50% for DNAH6 and 60% for DNALI1) or severely abnormal with only several foci (50% for DNAH6 and 40% for DNALI1) in sperm from *Cfap57^M/M^* mice when compared with those from *Cfap57^+/M^* mice (Figure 5, A and B). Surprisingly, DNAH10, a double-headed IDA protein, is still present on the mutant sperm flagella with a discretely distributed pattern, just like those ODA proteins (Supplemental Figure 6E). IDAs are divided into 7 types, 1 double-headed IDA and 6 single-headed IDAs. Different IDAs need different scaffolds and chaperone recruitment proteins for their formation, although they share a highly conserved structure (40). The more affected signals of DNAH6 and DNALI1, the single-headed IDA markers, and the less affected signals of DNAH10, the double-headed ODA marker, in sperm from *Cfap57^M/M^* mice revealed that single-headed IDAs were more significantly affected than double-headed IDAs in *Cfap57^M/M^* mouse sperm (Supplemental Figure 6F). Overall, our results suggested that CFAP57 played an essential role particularly in single-headed IDA assembly (Supplemental Figure 7).

### CFAP57 is essential for the assembly of IDAs.

We next explored whether the loss of IDA signals on flagella was due to reduced synthesis in the testes or assembly failure in the sperm flagella of the related proteins. The expression of DNAH6 and DNALI1, as well as another IDA marker, DNAH1, was assessed using immunoblotting in mouse testis lysates, using GAPDH and the ODA protein DNAI1 as loading controls. In *Cfap57^M/M^* mouse testes, these IDA proteins were expressed at levels comparable to those in *Cfap57^+/+^* and *Cfap57^+/M^* mice (Figure 5C), indicating that all these IDA proteins were normally synthesized in *Cfap57^M/M^* testes. Thus, we think that loss of long transcript-encoded CFAP57 protein interfered with the normal assembly of IDAs, resulting in the loss of IDAs in human and mouse sperm flagella.

## Discussion

In this study, we identified 2 stop-gain mutations in *CFAP57* (c.2872C>T, p. R958*; and c.2737C>T, p. R913*) that were recessively cosegregated with male infertility in 3 Pakistani families. Semen analysis of affected men revealed typical MMAF phenotypes. Both *CFAP57* mutations have been verified to cause loss of long transcript-encoded CFAP57 protein in spermatozoa from MMAF-affected individuals or from a *Cfap57*-mutant mouse model. Extensive analysis using *Cfap57^M/M^* mice revealed that loss of the long transcript of *Cfap57* disrupted IDA assembly in sperm flagella and single-headed IDAs were more likely to be affected. Our study provides direct evidence for the pathogenicity of *CFAP57* loss-of-function mutations in MMAF-affected individuals and determines an essential role for the long transcript-encoded CFAP57 in spermiogenesis in humans and mice.

IDAs were demonstrated to be vital for the motility of cilia or flagella. Pathogenic mutations in IDA genes cause MMAF phenotypes in humans, such as single-headed IDA genes *DNAH1* and *DNAH6* (4, 28) as well as double-headed IDA genes *DNAH2* and *DNAH10* (10, 27). Few studies have focused on the factors responsible for IDA assembly in male infertility (12). FAP57, the ortholog of CFAP57 in *Chlamydomonas*, is necessary for the assembly of a subset of IDAs, as it attaches to the microtubule near the IDA in cilia and flagella (41). The deletion of FAP57 led to a reduction in dynein g/d and associated dynein heavy chain proteins such as DHC3, DHC7, and DHC2. The cryo-ET results supported this conclusion and revealed that FAP57 was located at the base of the IDA (41). We demonstrated for the first time to our knowledge that CFAP57 is important for the normal localization of single-headed IDA in sperm flagella and is essential for male fertility. Our results corroborate the findings in *Chlamydomonas*, suggesting that CFAP57 function is highly conserved across species.

It is noteworthy that pathogenic mutations in corresponding IDAs or IDA assembly genes cause not only the loss of IDAs but also severely disorganized axonemal structures in sperm flagella in humans and mice (4, 10, 12, 27, 28, 42). Ultrastructural analysis of the sperm flagella from *CFAP57*-mutated individuals revealed the presence of a normal 9 + 2 structure with only IDAs’ absence. However, the sperm flagella from *Cfap57^M/M^* mice have a more disorganized axonemal structure in most cross sections. Similar findings have also been reported for other IDA proteins. For instance, in humans, *DNAH1* mutations caused MMAF, with severely damaged ultrastructure in sperm flagella, while about 50% of sperm from *Dnah1*-knockout mice have normal morphology and unaffected ultrastructure in flagella (4, 43). In addition, organized axonemal structure along with loss of IDAs were observed in MMAF cases carrying *DNAH10* mutations, but the axonemal structure of *Dnah10^–/–^* or *Dnah10^M/M^* mice was totally disorganized (10). Moreover, we found that signals of double-headed IDA and ODA proteins were weak and discontinuous but still detectable in the sperm flagella of *Cfap57^M/M^* mice. Combined with the transmission electron microscopy (TEM) results, the abnormalities of double-headed IDAs and ODAs may be the consequence of the disorganized ultrastructure in sperm flagella from *Cfap57^M/M^* mice, resulting from the IDA assembly failure in *Cfap57^M/M^* mice. Therefore, it is supposed that the integrity of the axoneme structure in mutant mouse sperm is more susceptible to IDA assembly failure than in humans.

Previously, a pathogenic mutation in *CFAP57* (NM_001195831.2, c.1762C>T) was identified in a PCD-affected man, but his reproductive status was unknown (35). In our study, all individuals except P6 carrying homozygous *CFAP57* mutations claimed no PCD symptoms except asthenozoospermia. It should be noted that both mutations (M1 and M2) identified in our study only affected the long transcript of *CFAP57* (NM_001195831.2), while the previously reported mutation affected both transcripts (NM_001195831.2 and NM_152498.3) (Supplemental Figure 3A). Since the short transcript-encoded protein retained most of the WD repeat domains that are supposed to be important in cilium-associated functions (12, 44), it may reserve the function in cilia in MMAF-affected men with CFAP57 mutations (M1 and M2). This is further supported by the expression of the short transcripts in human nasal epithelial cells (Supplemental Figure 3, B–D). Besides, we have demonstrated the existence of short transcript of *Cfap57* in mice, which we believe was never recorded in any database before. This could explain why *Cfap57^M/M^* mice showed normal growth and development, while the knockout mice recorded in the Mouse Genome Informatics database (MGI:6152429) and the man harboring homozygous *CFAP57* mutation (c.1762C>T) displayed a severe PCD-like phenotype. Together, these results suggest that the long and short transcripts of CFAP57 have distinct functional roles, the long transcript is essential for the development of sperm flagella, and the short one is critical for the development of cilia, which is worth exploring further.

Our study provides a potentially novel direction for investigating the basis of structural heterogeneity between cilia and flagella. Although they have a high similarity in ultrastructure, the key IDA assembly factors, such as CFAP57, play essential roles in cilia and flagella through distinct transcripts from a single gene. Our study also suggests that different transcripts from one gene can be involved in different biological processes. When evaluating the specific effects of such gene mutations in human disorders, it is necessary to comprehensively consider the effects of mutations on all transcripts of the gene.

In conclusion, our study based on MMAF-affected individuals and the corresponding mutant mouse models demonstrates that homozygous loss-of-function mutations in *CFAP57* cause MMAF and male infertility in mice and humans due to the disruption of IDA assembly. Our findings highlight the essential and conserved role of *CFAP57* isoforms in cilia and flagella and improve our understanding of sperm flagellar anomaly etiology and pathophysiology. *CFAP57* can be used as a genetic screening marker in genetic counseling and diagnosis of MMAF and male infertility.

## Methods

### Participants.

In the present study, 3 Pakistani consanguineous families were recruited, with families 1, 2, and 3 having 3 (P1, P2, P3), 2 (P4, P5), and 2 (P6, P7) infertile brothers, respectively. Semen parameters, including semen volume, sperm concentration, and sperm motility, were analyzed at least twice for P2, P3, P6, and P7 in accordance with guidelines established by WHO (3). Morphological analysis was performed for P2 and P7 with at least 200 spermatozoa examined for each individual. All the patients answered a questionnaire regarding symptoms of PCD. The samples for fertile controls were obtained from volunteers at the First Affiliated Hospital of USTC.

### WES and bioinformatic analysis.

Total genomic DNA was isolated from peripheral blood of individuals of family 1 (III:1, III:2, and IV:6), family 2 (II:2 and II:4), and family 3 (III:1, III:2, IV:3, and IV:9) using the FlexiGene DNA Kit (QIAGEN) and fragmented using a Covaris Focused-ultrasonicator. Whole-exome capture and sequencing were performed using AIExome Enrichment Kit V1 (iGeneTech) and HiSeq 2000 platform (Illumina) following standard procedures. The reads were aligned to the human genome reference assembly (hg19) using BWA-MEM, and Picard software was used to remove PCR duplicates. DNA sequence variants were called using the Genome Analysis Toolkit HaplotypeCaller. Variants were annotated using ANNOVAR (45), and candidate pathogenic variant filtration was performed as we previously described (46–49). Details are shown in Supplemental Figure 2, Supplemental Table 1, and Supplemental Table 2. Bcftools (50) was used to detect runs of homozygosity. Runs of homozygosity over 1.5 Mb were used to calculate the inbreeding coefficients with an in-house script. The relatedness of family members was checked using Peddy (51). The sequences of the primers used for Sanger sequencing are listed in Supplemental Table 3.

### Generation of the polyclonal anti-CFAP57 antibody.

A CFAP57 polyclonal antibody was generated in rabbits using aa 701–900 of human CFAP57 (UniProt accession Q96MR6) as antigens by Dia-an Biotechnology.

### Cell culture and transfection.

HEK293T (ATCC) cells were cultured as we previously described (52) and transfected with P-N1 plasmids expressing mCherry-tagged human and mouse CFAP57 using Lipofectamine 3000 (Invitrogen, Thermo Fisher Scientific, L3000015) according to the manufacturer’s instructions. Cells were harvested for immunoblotting 24 hours posttransfection. The sequences of the primers used are listed in Supplemental Table 3.

### Immunoblotting.

Immunoblotting was performed as we previously described (26, 49, 53). Briefly, cultured cells were lysed using Bolt LDS Sample Buffer (Invitrogen, Thermo Fisher Scientific, B0008) with NuPAGE Antioxidant (Invitrogen, Thermo Fisher Scientific, NP0005) and boiled for 10 minutes. The protein extract from testes was prepared using lysis buffer (50 mM Tris, 150 mM NaCl, 0.5% Triton X-100, and 5 mM EDTA, pH 7.5) containing a protease inhibitor cocktail (Solarbio, P6730). Then the proteins were separated by SDS-PAGE and transferred onto nitrocellulose blotting membranes with a pore size of 0.45 μm (GE Healthcare, 10600002). For blocking, the membranes were incubated with TBS-Tween (TBST) buffer (50 mM Tris, 150 mM NaCl, and 0.5% Tween-20, pH 7.4) containing 5% nonfat milk for 30 minutes and then incubated with primary antibodies diluted in TBST buffer containing 5% nonfat milk at 4°C overnight. Incubation with secondary antibodies for 1 hour was followed by chemiluminescence development (ImageQuant LAS 4000, GE Healthcare). Additional antibodies are listed in Supplemental Table 4.

### Immunofluorescence staining.

The preparation of human semen smears and immunofluorescence staining were conducted as we described (49). The smears were fixed in 4% paraformaldehyde (PFA) for 4 minutes and then washed twice with PBS for 5 minutes, followed by permeabilization with 0.2% Triton X-100 in PBS for 30 minutes, followed by blocking with 3% skim milk. The smears were then incubated with primary antibodies at 4°C overnight, followed by secondary antibodies at 37°C for 1 hour. The antibodies used are listed in Supplemental Table 4.

### Generation of the Cfap57-mutant mouse model.

*Cfap57^M/M^* mice were generated by using CRISPR/Cas9 technology (54). Briefly, guide RNAs (gRNAs) targeting exon 18, which is close to the mutation sites in the infertile cases, were transcribed in vitro (Invitrogen, Thermo Fisher Scientific, AM1908). Then, the gRNAs and the Cas9 protein were electroporated into C57BL/6 mouse zygotes. Sanger sequencing and PAGE analysis were used to determine the genotypes of the pups. The sequence of the gRNA is 5′-TCATAAATTCGCTTCTCCTGG-3′, and the genotyping primers are given in Supplemental Table 3. All animal experiments were approved by the Institutional Animal Care and Use Committee of the USTC and conducted according to the committee guidelines.

### Histology.

The epididymides and testes were harvested and fixed in Bouin’s fixative solution. As described previously (55), tissues were dehydrated using a graded series of ethanol followed by embedding in paraffin and serial sectioning, followed by H&E staining. Using a light microscope (Nikon) equipped with a charge-coupled device camera (Nikon), slides were examined, and images were captured.

### Analyses of mouse sperm count, morphology, and motility.

Eight-week-old mice were sacrificed by cervical dislocation. As described previously (49), we assessed sperm number by cutting the epididymis into pieces in PBS and incubating them for 30 minutes at 37°C to release the sperm. A hemocytometer was used to count the sperm released from the epididymis under a microscope. For sperm motility, the epididymis was cut and placed in human tubal fluid containing 10% FBS at 37°C for 5 minutes, after which the liquid containing sperm was collected, and sperm motility was determined using CASA. To examine sperm morphology, slides were fixed in 4% PFA for 4 minutes, washed with PBS, and stained with H&E. At least 200 spermatozoa from each mouse were examined to determine the percentages of morphologically abnormal spermatozoa.

### Electron microscopy analyses.

Spermatozoa were fixed in 0.1 M phosphate buffer (pH 7.4) containing 4% glutaraldehyde, 4% PFA, and 0.2% picric acid at 4°C for at least 8 hours. SEM and TEM assays were conducted as we previously described (23, 56).

### Statistics.

Statistical analysis was performed using GraphPad Prism 6 (GraphPad Software). Values in tables and graphs are expressed as mean ± SD. Two groups of mice were compared with Student’s 2-tailed *t* test for independent data. *P* < 0.05 was considered significant.

### Study approval.

The USTC Ethical Committee approved this study. An informed consent form was obtained from each participant before the study began.

## Author contributions

AM, BZ, and HM designed the experiments; AM, JC, XH, and BZ performed the experiments; AM wrote the manuscript; HA, TA, IA, ZM, SD, WS, BS, GM, FI, MAK, and AK recruited the families, performed semen analysis, and collected samples; QS, ZM, HZ, HM, LW, BX, and QL modified the manuscript; JZ, DZ, YZ, and HZ performed the WES sequencing and WES data analysis; and QS and HZ conceived and supervised the study and article drafting. All authors contributed to the report.

## Supplementary Material

Supplemental data

Supplemental table 1

Supplemental table 2

Supplemental table 3

Supplemental table 4

Supplemental video 1

Supplemental video 2

## Figures and Tables

**Table 1:**
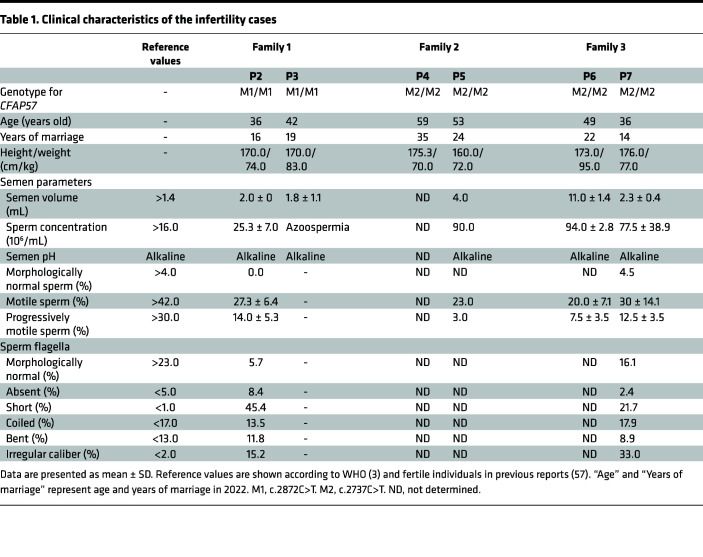
Clinical characteristics of the infertility cases

**Table 2:**
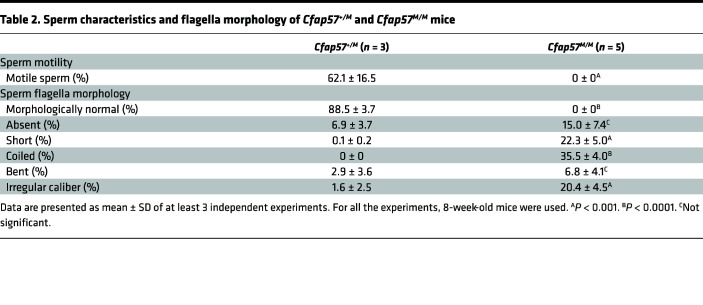
Sperm characteristics and flagella morphology of *Cfap57^+/M^* and *Cfap57^M/M^* mice

## Version 1 02/08/2023

Electronic publication
